# Yield and Grain Quality of Common Wheat (*Triticum aestivum* L.) Depending on the Different Farming Systems (Organic vs. Integrated vs. Conventional)

**DOI:** 10.3390/plants12051022

**Published:** 2023-02-23

**Authors:** Katarzyna Mitura, Grażyna Cacak-Pietrzak, Beata Feledyn-Szewczyk, Tomasz Szablewski, Marcin Studnicki

**Affiliations:** 1 Department of Food Technology and Assessment, Institute of Food Sciences, Warsaw University of Life Sciences, Nowoursynowska, 159C Street, 02-776 Warsaw, Poland; 2 Department of Systems and Economics of Crop Production, Institute of Soil Science and Plant Cultivation—State Research Institute, Czartoryskich 8 Street, 24-100 Pulawy, Poland; 3 Department of Food Quality and Safety Management, Faculty of Food Science and Nutrition, Poznan University of Life Sciences, Wojska Polskiego 31 Street, 60-624 Poznan, Poland; 4 Department of Biometry, Institute of Agriculture, Warsaw University of Life Sciences, Nowoursynowska 159 Street, 02-776 Warsaw, Poland

**Keywords:** spring wheat, cultivars, farming system, grain yield, 1000 grain weight, protein content, wet gluten, falling number

## Abstract

Genotype (cultivar), soil and climatic conditions, the agrotechnology used, and the interaction of the factors mentioned play a key role in the yield and quality of wheat grain. Currently, the European Union recommends the balanced use of mineral fertilisers and plant protection products in agricultural production (integrated production) or the use of only natural production methods (organic production). The aim of the study was to compare the yield and grain quality of four spring common wheat cultivars Harenda, Kandela, Mandaryna, and Serenada, grown under three farming systems: organic (ORG), integrated (INT), and conventional (CONV). A three-year field experiment was conducted between 2019 and 2021 at the Osiny Experimental Station (Poland, 51°27′ N; 22°2′ E). The results showed that significantly the highest wheat grain yield (GY) was obtained at INT, while the lowest was obtained at ORG. The physicochemical and rheological characteristics of the grain were significantly influenced by the cultivar factor and, with the exception of 1000 grain weight (TGW) and ash content (AC), by the farming system. There were also numerous interactions between the cultivar and farming systems, which suggests different performances of cultivars and, in fact, that some cultivars are better or worse suited to different production systems. The exceptions were protein content (PC) and falling number (FN), which were significantly highest in grain with CONV and lowest in grain with ORG farming systems.

## 1. Introduction

Common wheat (*Triticum aestivum* L.) is one of the most widely grown cereal crops [1,2]. In the last three seasons (2018–2021), the global wheat acreage was 213.9–219.0 million ha and grain yields ranged from 732.1 to 760.9 million tonnes. The largest global wheat producers in the 2020–2021 season were China (134.3 million tonnes), India (107.6 million tonnes), Russia (85.9 million tonnes), the USA (49.7 million tonnes), Canada (35.2 million tonnes), France (30.1 million tonnes), and Ukraine (24.9 million tonnes). Wheat is also the most important cereal crop in the EU, including in Poland [2,3]. In 2018–2022, the acreage under wheat cultivation in Poland was 2.4–2.5 million hectares, with harvests ranging from 8.6 million tonnes in the 2018–2019 season to 12.4 million tonnes in the 2020–2021 season [2].

Wheat, due to its valuable chemical composition of the grain and its exceptional technological properties, is a basic cereal for food processing, referred to as a so-called bread cereal in many countries [4,5]. Wheat grain is a source of carbohydrates, protein, dietary fibre, and fat, as well as minerals (including P, K, Ca, and Mg), B vitamins, and other bioactive substances [6].

Wheat should be cultivated in a way that ensures a high grain yield of adequate quality to meet the requirements of food processing [7]. Wheat grain yield and quality are determined by many factors, including genotype (cultivar), habitat conditions (soil and climate) and agricultural practices [8,9,10,11,12,13,14,15,16,17,18,19,20,21,22,23]. These factors also affect the economic viability of production and the provision of food security [24,25,26,27]. By 2050, the world population is projected by the United Nations to grow to about 10 billion [28], so it is important to pay attention to the possibilities of meeting the growing global food demand for an ever-increasing population [3].

Nowadays, wheat cultivation is mostly carried out, especially in highly developed countries, in an intensive way (the conventional system), which is based on the extensive use of mineral fertilisers and chemical plant protection products [24]. Such a production system ensures high yields [24,29,30,31], but is associated with many risks, especially to the environment [32]. Due to the need to reduce the harmful impact of agriculture on the environment, the use of less intensive production methods is currently one of the priorities of the European Union’s agricultural policy (European Green Deal), which recommends the sustainable use of mineral fertilisers and plant protection products in agricultural production (the integrated system) or the use of only natural methods (the organic system). One of the main objectives of this strategy is to increase the organic farming area to 25% of the total agricultural area in the EU by 2030 [3,33]. It is worth emphasising that at present, economic considerations, i.e., increasing prices for mineral fertilisers and plant protection products due to, among other things, the political situation (Russian–Ukrainian war), are also forcing agricultural producers to introduce restrictions on the use of industrial inputs [34].

The aim of this study was to determine the effect of a farming system on the yield and grain quality of four spring cultivars of common wheat. It should be emphasised that three farming systems, organic (ORG), integrated (INT), and conventional (CONV), were included in our study, whereas most studies conducted to date have been limited to comparing organic and conventional systems only. The scientific hypothesis assumed that with less intensive agrotechnology it is possible to obtain a high yield of wheat grain of a quality that meets the requirements of food processing.

## 2. Results and Discussion

### 2.1. Grain Yield

**Figure 1:**
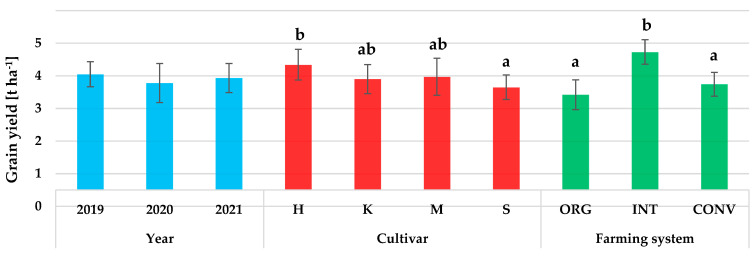
The effect of year, cultivar, and farming system on grain yield (GY). Different letters correspond to significant differences (α = 0.05) between means according to Tukey’s test. Error bars indicate the standard error of the mean. Abbreviations: H—Harenda, K—Kandela, M—Mandaryna, S—Serenada, ORG—organic, INT—integrated, and CONV—conventional.

The grain yield (GY) was significantly influenced by the genotype (cultivar) and farming system (Figure 1). The weather conditions during the years of the study did not significantly affect the wheat yield; nevertheless, variation in yield was observed between the years of the study. The highest GY (mean 4.05 t ha^−1^) was obtained in 2019, which experienced more favourable weather conditions, especially at the tillering stage of wheat (April) (Figure 7, Table 5). Favourable weather conditions also prevailed in May and June. These months had high rainfall, which is considered particularly important for wheat yield [35]. In contrast, the least favourable conditions at the tillering stage were in 2020, due to the exceptionally high drought during this period. The GY in 2020 averaged 3.78 t ha^−1^ and was about 9% lower than in 2019. The influence of weather conditions, primarily the amount and distribution of precipitation, on spring wheat yield has been indicated by the results of studies conducted by many authors [11,14,15,16,22,35,36,37,38,39,40]. Spring wheat reacts particularly unfavourably to water deficiency during the tillering period at the stem [41], which was partially confirmed in our study.

Our own research showed that GY was significantly influenced by the cultivar factor (Figure 1). The highest yielding wheat cultivar was Harenda (mean 4.34 kg ha^−1^). Compared to this cultivar, the cultivar Serenada (mean 3.65 kg ha^−1^) had a significantly lower yield. The GY of wheat cultivars Kandela and Mandaryna was at a similar level (mean 3.90 and 3.97 kg ha^−1^, respectively). In general, the GY of the wheat cultivars tested was quite low, typical of the spring form. Spring wheat has a lower yield potential than winter wheat due to the shorter growing season [42,43]. The cultivar variation in the spring wheat yield shown in our study has also been indicated by the results of previous studies [14,15,39,40,44,45,46].

**Figure 2:**
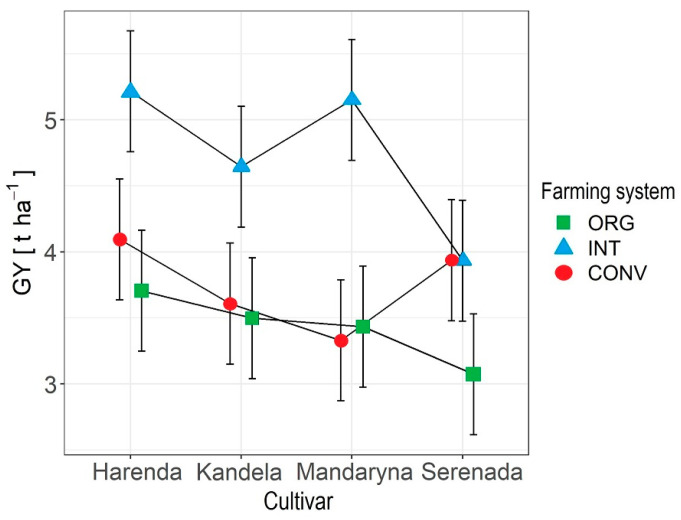
The interaction effects of cultivar and farming system on GY.

The GY of wheat depended significantly on the farming system and the interaction between the cultivar and farming system (Figure 1 and Figure 2). Significantly, the highest GY was obtained in the INT farming system (mean 4.73 kg ha^−1^); wheat yields in this system were higher than in CONV and ORG farming systems, by 20.9% and 27.7%, respectively (means: 3.74 and 3.42 kg ha^−1^). Lower wheat yields in the ORG rather than the CONV farming system are indicated by the results of Váňová et al. [9] and Billsborrow et al. [30]. On the other hand, Kuś et al. [39] showed a lower yield potential of wheat in the ORG system than the INT farming system. The literature data [11,15,36,38] suggest that wheat yield is strongly influenced by the intensity of the agrotechnology used in the crop, primarily NPK fertilisation and crop protection, which explains the lower wheat grain yields from organic farming, where the use of mineral fertilisers and chemical plant protection products is prohibited [40]. In our research, the application of an increased level of agrotechnology (CONV farming system) did not increase the yield of any of the tested wheat cultivars compared to the INT farming system and in the case of the Mandaryna cultivar, also compared to the ORG farming system. The results obtained indicate that for each of the wheat cultivars tested, the most optimal in terms of achieving high grain yields is to cultivate in the INT farming system, which is less costly and more environmentally friendly than the high-input CONV system. 

### 2.2. Physical Properties 

The hectolitre weight (HW) depended significantly on the weather conditions in the years of the study, genotype (cultivar), and farming system (Table 1). The significantly highest HW was characteristic of the grain from the 2019 harvest (mean 77.9 kg hl^−1^), the value of this parameter being 5.8% higher than in 2020 and 8.9% higher than in 2021 (means: 73.4 and 71.0 kg hl^−1^), respectively. The influence of weather conditions on the HW of wheat grain was also shown by Bilsborrow et al. [30] and Švančárková et al. [47], while no such correlations were found in the study of Cacak-Pietrzak [48]. 

**Figure 3:**
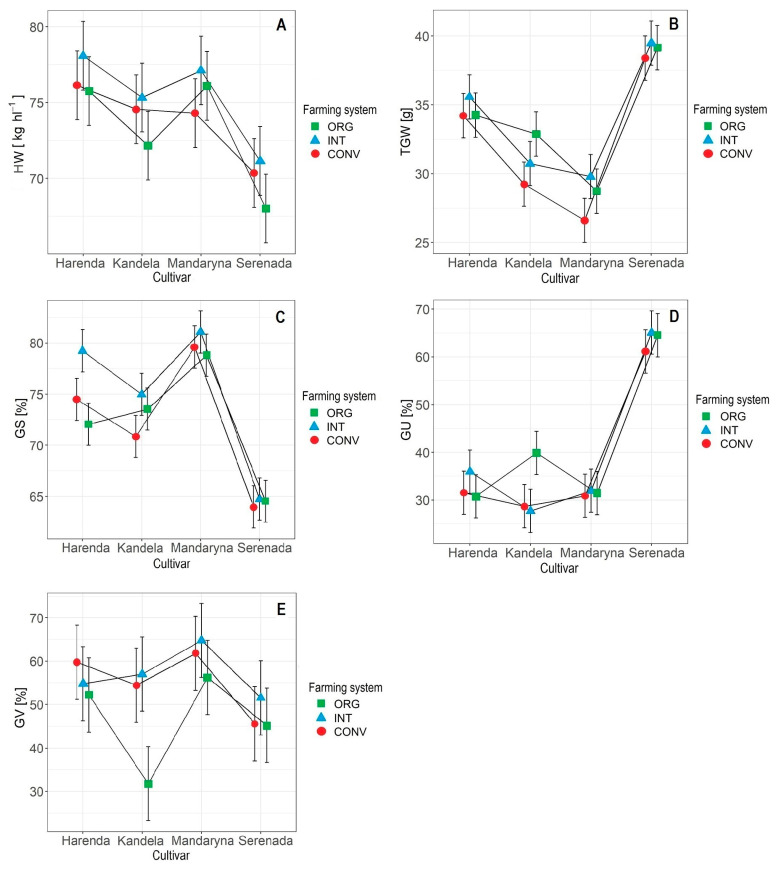
The interaction effects of cultivar and farming system on the physical properties of wheat grain. Abbreviations: (**A**) HW—hectolitre weight, (**B**) TGW—1000 grain weight, (**C**) GS—grain selectivity, (**D**) GU—grain uniformity, (**E**) GV—grain vitreousness, ORG—organic, INT—integrated, and CONV—conventional.

Among the tested wheat cultivars, the grain with the highest HW was found in the Harenda cultivar (mean 76.7 kg hl^−1^) (Table 1). A significantly lower HW compared to the other cultivars was characteristic of the wheat grain of the Serenada cultivar (mean 69.8 kg hl^−1^). The cultivar variation in the HW of wheat grain shown in our study has also been indicated by other authors [9,37,48,49]. The HW depended significantly on the farming system used in the crop and the interaction between the cultivar and the farming system (Table 1, Figure 3A). Significantly, the highest HW was characteristic of the grain with the INT farming system (mean 75.6 kg ha^−1^). The HW values of grain from ORG and CONV farming systems were similar (mean 73.0 and 73.8 kg hl^−1^, respectively). Similar correlations were obtained by Dziki et al. [9]. On the other hand, the lack of a significant effect of the ORG and CONV farming systems on wheat HW has been indicated by the results of Billsborrow et al. [30], Cacak-Pietrzak [48], and Sobolewska and Stankowski [49]. The response of cultivars to the applied growing conditions varied. The highest HW was characteristic of the grain under INT, and the lowest, with the exception of the cultivar Mandaryna, was characteristic of the grain under the ORG farming system. 

According to PN-R-74103 [50], the HW of wheat grain intended for food processing should be no less than 72.0 kg hl^−1^. This requirement, irrespective of the farming system used in cultivation, was met by the wheat grain of the Harenda, Kandela, and Mandaryna cultivars. On the other hand, the wheat grain of the Serenada cultivar from all three farming systems was characterised by a HW lower than the required one.

The 1000 grain weight (TGW) depended significantly on the genotype (cultivar), but this parameter was not influenced by weather conditions or the farming system (Table 1). In the studies conducted by Sułek et al. [16] and Cacak-Pietrzak [48], the course of weather significantly affected the TGW of wheat, while our own research did not confirm such a relationship. Such a relationship was also not found by Dziki et al. [9] or Rozbicki et al. [11].

The cultivar Serenada (mean 39.0 g) was characterised by the significantly highest TGW (Table 1) which, at the same time, had the significantly lowest HW. The significantly lowest TGW was characteristic of the cultivar Mandaryna, whose TGW (mean 28.4 g) was 27.2% lower than that of the cultivar Serenada. In general, with the exception of the Serenada cultivar, TGW values were low, but were typical of the spring form of wheat [46]. Spring wheat is characterised by a lower TGW than winter wheat, due to its shorter growing season [42,43,46]. The cultivar differences in wheat TGW found in our research have also been indicated by the results of many authors [8,9,10,11,15,16,39,48,51,52,53].

Significant interactions were found between the cultivar and farming system for TGW wheat grain (Figure 3B). The highest TGW, with the exception of the cultivar Kandela, was distinguished by grain from the INT farming system. For all wheat cultivars tested, grain from the CONV farming system was characterised by the lowest TGW. Lower TGW values of wheat from cultivation in the CONV rather than the ORG farming system have also been indicated by the results of Váňová et al. [8] and Marzec et al. [10]. In turn, Dziki et al. [9], Sobolewska and Stankowski [49], and Mazurkiewicz [54] showed that the TGW of wheat from ORG was significantly lower than in the CONV farming system, while in the studies of Cacak-Pietrzak [48] and Mazzocini et al. [31], no effect of farming system on this parameter was found. 

The TGW is one of the parameters that determines the suitability of wheat grain for milling. The higher the value of this parameter, the higher the yields of low-extraction (light) flour that can be obtained. This is due to the higher proportion of flour endosperm in the grain. There are no mandatory requirements for the minimum value of this parameter, but the higher the TGW, the better the potential milling properties of the grain [5]. In our study, the highest TGW was distinguished by the cultivar Serenada.

Grain selectivity (GS) and grain uniformity (GU) parameters were significantly influenced by the genotype (cultivar) and the farming system, while no effect of years on either parameter was shown (Table 1). A significant effect of weather conditions on GS and GU was indicated by the results of earlier studies with winter wheat [48]. 

Significant cultivar variation in GS and GU was found in our study (Table 1). Significantly, the highest GS was characteristic of the wheat grain of the Serenada cultivar (mean 64.4%), which could be associated with the high TGW of this wheat cultivar. The GS values of the other wheat cultivars were about two times lower (mean 31.4–36.1%), which was due to the low TGW of these wheat cultivars. The low GS of spring wheat grain has also been indicated by the results of Marzec et al. [10]. In our experiment, the wheat of the Serenada cultivar was the only one characterised by the same GS and GU values, which indicates at the same time high grain selectivity and uniformity. Significantly, the highest GU was characteristic of grains of the Mandaryna cultivar (mean 79.8%). The cultivar variation in the GS and GU of wheat found in our study has also been indicated by the results of Mäder et al. [13], Feledyn-Szewczyk et al. [52], and Kwiatkowski et al. [55]. In contrast, a study by Cacak-Pietrzak [48] showed a significant effect of genotype only on winter wheat GS.

We also observed a significant effect of the farming system and the interaction between the cultivar and the farming system on GS and GU (Figure 3C,D). Significantly, a higher GS was characteristic of the wheat grain of ORG and INT (means: 41.6% and 40.2%, respectively) rather than the CONV farming system (mean 38.1%). The response of the cultivars to the applied growing conditions was uneven. The highest GS value was obtained in the INT farming system. Harenda and Mandaryna cultivars had the lowest GS with the ORG farming system, while Kandela and Serenada cultivars had the lowest GS with the CONV farming system. The highest GU was characteristic of the grain from the INT farming system, with the exception of the Kandela cultivar, which had the highest GU in the ORG farming system. Grain with the lowest GU was obtained from the CONV farming system in the Mandaryna and Serenada cultivars, from the INT farming system in the Kandela cultivar, and from the ORG farming system in the Harenda cultivar. 

Grain selectivity and grain uniformity are important parameters affecting the yield obtained during the milling of low-extraction (light) flour, which is why these parameters are often used in the milling industry to assess the milling suitability of wheat grain [5]. There are no strict requirements, but the higher the values of these parameters, the better the grain is considered to be for milling purposes. 

The grain vitreousness (GV) of wheat was significantly influenced by the year, the genotype (cultivar), the farming system, and their interaction (Table 1, Figure 3E). The significantly highest GV was characteristic of the grain harvested in 2020 (mean 63%), while the significantly lowest GV was that of the grain harvested in 2021 (mean 41%). A significant effect of weather on the GV of wheat has also been indicated by studies by Cacak-Pietrzak [48] and Branković et al. [56]. The cultivars Mandaryna and Harenda had the significantly highest GV (means: 61% and 56%, respectively). The GV of Serenada and Kandela cultivars averaged 47–48%. The significant effect of wheat cultivar on GV has also been indicated by studies by other authors [9,10,48,51,52,54]. 

Significantly, the lowest GV was characteristic of the wheat grain from the ORG farming system (mean 46%); the GVs of the grain from the other two farming systems were at similar levels (means 55–57%) (Table 1). The obtained correlations have been confirmed by the studies of other authors [9,10,54], which have shown that wheat grain from the CONV farming system was characterised by a higher proportion of vitreous grains than grain from the ORG farming system. The higher proportion of vitreous grains in the INT and CONV rather than the ORG farming system should be associated with a favourable effect on this trait of mineral fertilisation, especially nitrogen fertiliser [54]. Marzec et al. [10] showed that grain from intensive cultivation is characterised by higher endosperm vitreousness than grain from organic cultivation due to a higher content of protein substances that form a matrix closely surrounding the starch grains. In our study, there were interactions between the cultivar and the farming system (Figure 3E). The cultivars Kandela, Mandaryna, and Serenada had the highest GV of the grain from the INT farming system, while the cultivar Harenda had the highest GV from the CONV farming system. Cultivation in the ORG farming system resulted in the lowest GV of each of the wheat cultivars tested, which was particularly evident for the Kandela cultivar.

The vitreousness of the wheat grain is related to the chemical composition, and in particular to the ratio of protein to starch. Vitreous grain is characterised by a slightly higher protein content than flour grain and a tighter endosperm structure, which is why its grinding is more energy-intensive. During the milling of vitreous grain, larger amounts of flour are obtained in the final stage of milling. Such flours are characterised by low ash content and light colour [5]. The tested wheat varieties were characterised by an average share of vitreous grain.

### 2.3. Chemical and Rheological Properties 

**Figure 4:**
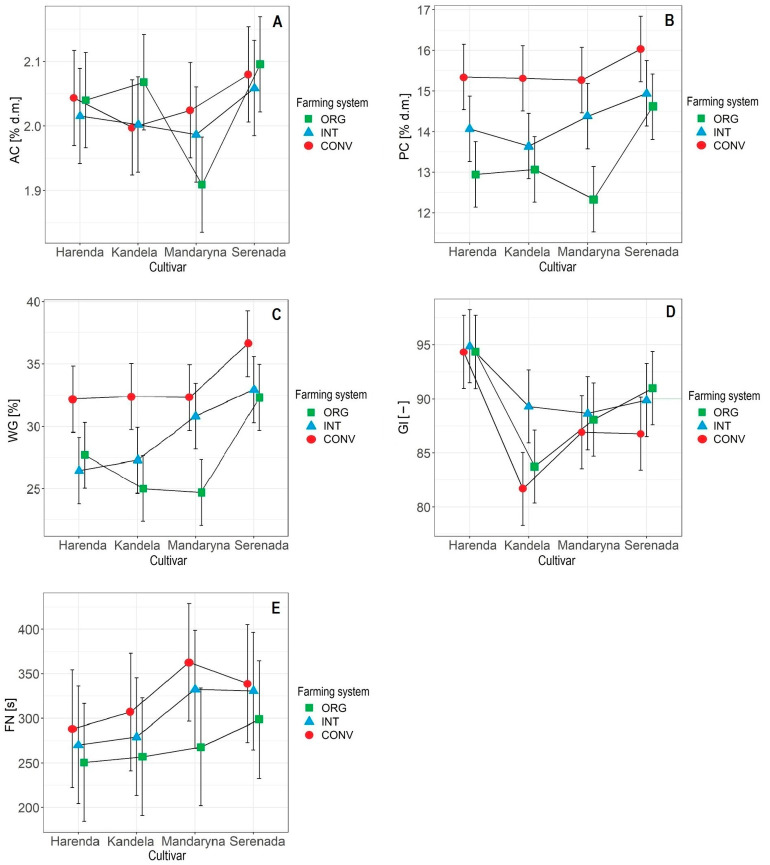
The interaction effects of cultivar and farming system on the chemical and rheological properties of wheat grain. Abbreviations: (**A**) AC—ash content, (**B**) PC—protein content, (**C**) WG—wet gluten, (**D**) GI—gluten index, (**E**) FN—falling number, ORG—organic, INT—integrated, and CONV—conventional.

The ash content (AC) in wheat grain was significantly influenced by genotype (cultivar), while no significant effect of the other sources of variation was found (Table 2, Figure 4A). A significant effect of weather conditions on the AC in wheat grain was also not shown by Mazzoncini et al. [57]. On the other hand, the effect of weather conditions on mineral accumulation in wheat grain has been indicated by the results of Cacak-Pietrzak [48] and Kihlberg et al. [58].

The significantly highest AC was characteristic of the grain of the cultivar Serenada (mean 2.08% d.m.), and the significantly lowest was characteristic of the grain of the cultivar Mandaryna (mean 1.97%. d.m.) (Table 2). In the grain of the other wheat cultivars (Harenda and Kandela), the AC was at the same level (mean 2.03% d.m.). The cultivar differentiation in wheat grain in terms of AC shown in our study has also been indicated by numerous authors [4,10,48,51,58]. The AC in the grain of all of the tested wheat cultivars was relatively high, but typical for the spring form [59]. Spring wheat contains more AC than winter wheat, due to the lower kernel weight and the resulting poorer proportion between the proportion of endosperm and seed coat, in which large amounts of mineral nutrients (ash) are accumulated [5].

In our experiment, there was no significant effect of farming system on AC in wheat grain, but there were interactions between the cultivar and the farming system (Table 2, Figure 4A). Similar relationships for winter wheat cultivars were obtained by Mazzoncini et al. [57]. In the studies of Toader et al. [60] and Cacak-Pietrzak [48], wheat grain from the ORG farming system was characterised by higher ash content than grain from the CONV farming system.

A reduction in AC in wheat grain with more intensive agrotechniques was also shown by Woźniak and Makarski [61]. The response of the tested wheat cultivars to the farming system used in the cultivation varied (Figure 4A). For the cultivars Harenda and Serenada, the lowest AC was characteristic of the grain with the INT farming system, for the cultivar Kandela it was with the CONV farming system, and for the cultivar Mandaryna it was with the ORG farming system. The highest ash content was characteristic of the grain with the ORG (cultivars Kandela and Serenada) or CONV farming systems (cultivars Harenda and Mandaryna).

The mineral (ash) content of wheat grain is an important quality parameter used when selecting grain for milling. Macro- and micronutrients are nutritionally important components [62]; however, too high of a content is undesirable due to an inferior milling value of the grain [5]. The ash content of wheat grain for production into low-extraction (light) flours should not exceed 1.85% d.m. [5]. The AC of the grain of each of the wheat cultivars tested, regardless of the production system, exceeded this value.

The protein content (PC) in wheat grain was significantly influenced by the year, genotype (cultivar), farming system, and their interaction (Table 2, Figure 4B). The grain with the highest PC significantly was obtained in 2021 (mean 15.2% d.m.), which was associated with a warm and humid April, favouring nitrogen accumulation in the plant, and with a warm June, which influenced the deposition of protein substances in wheat grain (milk maturity stage). In the wheat grain from the 2019 and 2020 harvests, the PC was similar (means: 14.0 and 13.8% d.m., respectively). The significant influence of weather conditions in shaping the amount of protein substances in wheat grain has also been indicated by the results of studies conducted by Sułek and Cacak-Pietrzak [15], Sułek et al. [16], Krejčířová [23], Polityko [38], and Cacak-Pietrzak [48]. The significantly highest PC was characteristic of the grain of the Serenada cultivar (mean 15.3% d.m.), in which the content of this component was higher by 1.0–1.1 p.p. than in the grain of the other wheat cultivars. Cultivar variability of wheat in terms of PC has been shown in many works [9,10,11,15,16,23,48].

Significantly the highest PC was characteristic of grain with the CONV farming system (mean 15.5% d.m.). In wheat grain from the INT and ORG farming systems, the mean PCs were, respectively, 14.3% and 13.2% d.m. According to Oleksy [63], the use of intensive technology in cultivation increases the PC in wheat grain, which was confirmed in our study. According to many authors [11,48,63,64], the main factor determining PC in wheat grain is the level of nitrogen fertilisation, but it should be emphasised that wheat uses mineral nitrogen more efficiently than organic nitrogen. The lower PC in wheat grain from the ORG compared to the INT and CONV farming systems shown in our study is, therefore, a consequence of the non-application of mineral nitrogen fertilisation in this farming system. The lower content of protein substances in wheat grain from the ORG rather than the CONV farming system has been indicated by the results of many studies [9,10,23,47,48,49,62,65]. In our research, the response of the cultivars to the farming system used in the crop was the same (Figure 4B). As the intensity of the agrotechnical level increased, the PC in the grain of each wheat cultivar increased linearly. It was the most visible in the case of the Mandaryna cultivar, while it was the least visible in the case of the Serenada cultivar.

PC is an important parameter used to assess the suitability of wheat grain when selecting the raw material for milling into baking flours. Wheat grain intended for this purpose should contain a minimum of 11.5% of this component [5,66]. It was found that the grain of each of the wheat cultivars tested, regardless of the farming system used in the cultivation, met the above quality requirement. 

The wet gluten (WG) content and gluten index (GI) values were significantly influenced by genotype (cultivar), farming system, and their interaction (Table 2, Figure 4C,D). Weather conditions in the years of the study only significantly affected gluten quality. No effect of weather conditions on WG content was also shown by Rozbicki et al. [11]. In contrast, the results of studies by Sułek et al. [15], Sułek and Cacak-Pietrzak [16], Krejčířová et al. [23], Holik et al. [36], Cacak-Pietrzak [48], and Keres et al. [64] indicate a significant effect of weather conditions on the accumulation of gluten proteins in wheat grain. Wheat grain from the 2019 and 2021 harvests had significantly higher GI values (mean 91) than grain harvested in 2020 (mean 86) (Table 2), but these differences, although statistically significant, were not technologically significant. A significant effect of weather conditions on GI values has also been indicated by Sułek and Cacak-Pietrzak [15], Sułek et al. [16], Ceseviciene et al. [37], and Cacak-Pietrzak [48], but Mazzoncii et al. [57], on the other hand, found no significant effect of weather on gluten quality. 

Significant cultivar variation was observed in WG content and GI values (Table 2). The significantly highest WG content was characteristic of the wheat grain of the Serenada cultivar (mean 34.0%). In the grain of the other cultivars, the WG content ranged from 28.2–29.3% and was not significantly differentiated. The wheat cultivar differences in WG content shown in our study have also been indicated by other authors [11,15,23,48,53].

The highest significant GI value was characteristic of the wheat grain of the Harenda cultivar (mean 95), and the lowest was characteristic of the Kandela cultivar (mean 85). Significant differences in the quality of gluten leached from the grain of different wheat cultivars have also been indicated by the results of Rozbicki et al. [11], Sułek et al. [15], Cacak-Pietrzak [48], and Sobolewska and Stankowski [49].

We also observed a significant effect of farming system on WG content and GI values (Table 2). The significantly highest WG content was characteristic of the wheat grain obtained from the CONV farming system (mean 33.4%), in which the content of this component was higher by 4 p.p. than in the grain with INT (mean 29.4%) and by 5.9 p.p. compared to grain with ORG (mean 27.5%). Similar relationships were shown by Sobolewska and Stankowski [49] in an experiment with winter wheat. The beneficial effect of an increase in the intensity of cultivation technology on WG content in wheat grain has also been indicated by numerous other authors [11,38,47,48,49]. The GI values of wheat grain from the ORG and INT farming systems were at the same level (mean 91). The significantly lower value of this index was characteristic of the grain from the CONV farming system (mean 87). The literature data [36,48,57] indicate a negative effect of production intensity on gluten quality, which was also confirmed by our own research. Fertilising wheat with too-high nitrogen doses generally leads to a reduction in gluten quality. According to Cacak-Pietrzak [48], this is due to an increase in the proportion of the low-particle fraction of gliadin in the protein. Sobolewska and Stankowski [49] showed no variation in the GI values of wheat grain with ORG and CONV farming systems.

We also found that WG content and GI values were also significantly influenced by interactions between the cultivar and the farming system (Figure 4C,D). For all of the wheat cultivars tested, the highest WG content was characteristic of the grain from the CONV farming system, which at the same time had the lowest GI values, confirming the inverse relationship between gluten quantity and quality shown in other studies [48,57]. The cultivars Kandela, Mandaryna, and Serenada had the lowest WG content with the ORG farming system, while Harenda had the lowest in the INT farming system. Kandela and Mandaryna had the highest GI values with INT and Serenada had it with the ORG farming system, while the production system did not differentiate the values of this indicator in Harenda grain.

The quantity and quality of gluten proteins are important parameters for assessing the suitability of wheat grain as a raw material to produce flours for baking purposes. The WG content in wheat grain should not be lower than 27% and the GI value should not be lower than 60 [48]. The requirement for WG content was not met by the grain of two wheat cultivars (Kandela—mean 25.0%, Mandaryna—mean 24.7%) grown in ORG and the cultivar Harenda grown in the INT farming system (mean 26.5%). Nevertheless, it was found that grain with a high WG content could also be obtained in the ORG farming system, as exemplified by the cultivar Serenada (mean 32.3%). On the other hand, the grain of all of the wheat cultivars tested, irrespective of the farming system used, met the gluten quality requirement.

The falling number (FN) values, an indicator for assessing α-amylase activity, were significantly influenced by weather conditions, genotype (cultivar), and farming system (Table 2). Significantly, the lowest amylolytic enzyme activity (mean FN 414 s) was characteristic of the grain harvested in 2020, which was due to the lower amount of rainfall during the maturation and harvesting of wheat from the field compared to the multi-year average (Figure 7). In the other two years of the study, amylolytic enzyme activity was higher than in 2020 (means FN 239–243 s), which can be explained by less favourable weather conditions during harvesting. Particularly unfavourable conditions for grain maturation and harvest occurred in August 2021, which saw higher rainfall compared to the multi-year average (Figure 7). The significant influence of weather conditions on amylolytic enzyme activity has been indicated by the results of many authors [11,15,16,48,67].

Significantly higher FN values were obtained for grain of the cultivars Mandaryna and Serenada (means: 321 and 323 s, respectively) than for grain of the cultivars Harenda and Kandela (means: 270 and 281 s, respectively) (Table 2). The influence of the cultivar factor on FN values has also been indicated by the results of studies conducted by Rozbicki et al. [11], Sułek et al. [16], and Sobolewska and Stankowski [49]. 

Significantly, the highest FN was characteristic of the grain with the CONV farming system (mean 324 s), while, significantly, the lowest was for the grain with the ORG farming system (mean 265 s) (Table 2). Similar results were obtained by Ingver et al. [65], while in a study by Sobolewska and Stankowski [49], a higher FN value was characteristic of the wheat grain grown in the ORG rather than the CONV farming system, and in a study by Cacak-Pietrzak [48], the farming system did not significantly affect this parameter. The response of the cultivars to the applied growing conditions was similar (Figure 4E). There was an increasing trend in FN values with increasing production intensification for all of the wheat cultivars tested.

Amylolytic enzyme activity is an important parameter used in the selection of grain for milling into baking flours. Too-low values of this parameter (below 150 s) indicate grain sprouting, which disqualifies its use for food processing. Grain intended for milling purposes should be characterised by an FN of at least 200–250 s [48]. This requirement was met by the grain of all of the wheat cultivars tested, irrespective of the farming system used in the cultivation.

### 2.4. PCA Analysis 

**Figure 5:**
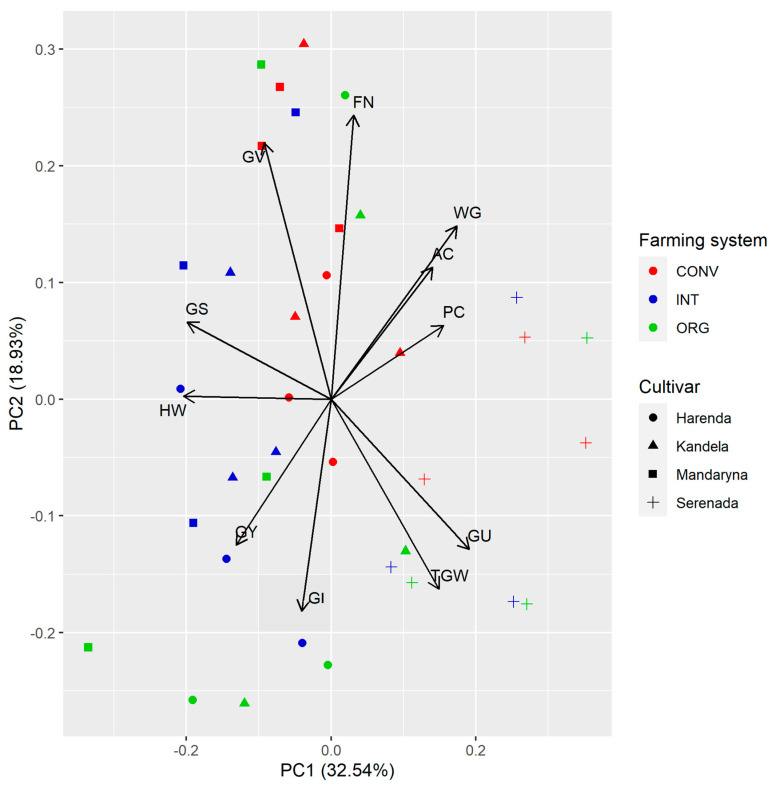
Biplot of PCA for all of the study traits. Abbreviations: HW—hectolitre weight, TGW—1000 grain weight, GS—grain selectivity, GU—grain uniformity, GV—grain vitreousness, AC—ash content, PC—protein content, WG—wet gluten, GI—gluten index, and FN—falling number.

Figure 5 presents the results of principal component analysis (PCA) for all of the study traits. We observe that the features GY, GS, and HW were strongly positively related with PC1, while they were negatively related with GU and TGW. GV and FN were positively related with PC2 and negatively related with GI. PC unfortunately did not show association with the first two principal components. The Serenada cultivar stands out here compared to the other three cultivars. It is characterised by a relatively low yield but at the same time a high content of PC and WG, regardless of the farming system in which it was grown.

## 3. Materials and Methods

### 3.1. Site Characteristics, Experimental Design, and Agronomic Practices 

**Figure 6:**
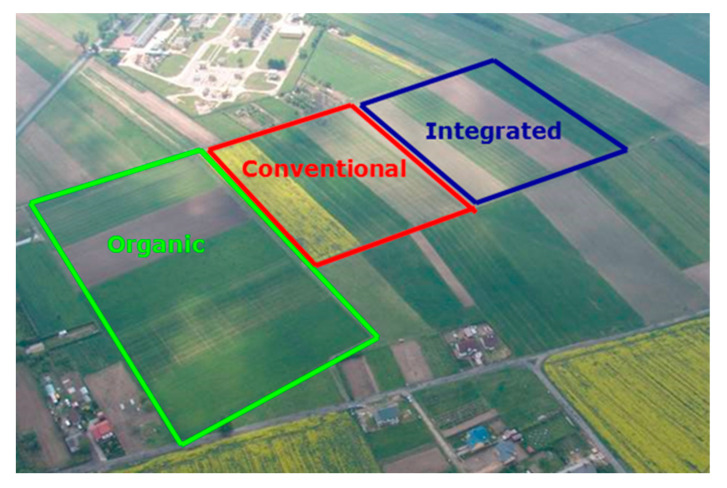
Scheme of the Osiny experimental field with a breakdown of the different farming systems.

A three-factor experiment with four spring wheat cultivars (Table 3) grown under three farming systems, ORG, INT, and CONV, was established in triplicate in 2019–2021. The field experiment was conducted at the Institute of Soil Science and Plant Cultivation—State Research Institute—at the Experimental Station in Osiny (Figure 6), located in the central-eastern part of Poland (51°27′ N; 22°2′ E), on a loamy soil (Halpic) with a loamy sand texture. The area of a single experimental plot was 30 m^2^, and the area for sowing and grain harvest was 25 m^2^. Agrotechnical treatments were carried out according to good agricultural practice at the optimum time for this region. The sowing rates were the same for each cultivar, amounting to 450 grains per m^2^. The row spacing was 12 cm and the sowing depth was 5 cm.

Crop rotations and agrotechnical treatments were suited to the tested farming systems: ORG, INT, and CONV (Table 4). In the ORG and INT farming systems, the potato was a forecrop for spring wheat, while in the CONV farming system, spring wheat was cultivated after the winter wheat due to the simplification of rotation in a high-input system. In the ORG farming system, the level of C_org_ in the soil was the highest, while the content of P and K was the lowest in comparison to the other systems, which can influence the grain yield and quality. Soil tillage was similar in all of the crop production systems; in general, it was a traditional plough system. Due to the conditions when assuming the experiment, the rows of spring wheat in the ORG have east–west orientation, while in the CONV and INT farming systems rows of spring wheat were north–south-oriented (Figure 6). The tested farming systems on arable land were characterised by different agricultural management systems (Table 4). In the ORG farming system, no synthetic pesticides or natural phosphorus (P) or potassium (K) fertilisers such as crude potassium salt or kainite, as well as compost, applied once in a crop rotation for potato (30 t ha^−1^), were applied. In the CONV farming system, crops were cultivated intensively, i.e., with high rates of synthetic mineral fertilisers and pesticides. In the INT farming system, balanced mineral and organic fertilisation (about 20–30% lower than in the CONV farming system), adaptation to the crop requirements, and soil fertility were used. Crop protection in compared farming systems is detailed in the Appendix A (Table A1). 

### 3.2. Meteorological Conditions

Weather data were obtained from the Agrometeorological Station located at the Osiny Experimental Station, where field experiments were conducted. The course of weather conditions was evaluated on the basis of monthly data: total precipitation (mm) and average air temperature (°C) measured 2 m above ground level. Weather conditions during the years of the field experiments (2019–2021) were compared to the long period average (1951–2021). In addition, in order to further define the hydrothermal conditions in the different growing seasons, the values of the Selyaninov hydrothermal index (k) were calculated and compared against the limits presented by Skowera [68]. 

Weather conditions varied during the years of the field experiments (2019–2021). In 2019, there were favourable weather conditions in March (rainfall 22.7 mm, mean daily air temperature 5.5 °C). April, based on the value of the Selyaninov hydrothermal coefficient, was assessed as being quite dry (Table 5). The total precipitation in April (35 mm), with a high for this period’s average daily temperature (9.6 °C) (Figure 6), caused water deficiency in the initial growth phases, which in the tillering phase results in the inhibition of the development of aboveground parts and roots, as well as a reduction in the number of stalks, ears, and spikelets per ear. Rainfall totals in May and June are of great importance in shaping yield. In May 2019, the rainfall total was 86.1 mm. Based on the value of the Selyaninov hydrothermal coefficient, weather conditions in this month were defined as being humid. The sum of precipitation in June and July was twice as high as the sum of precipitation from the long period (1951–2021). The highest daily air temperatures were recorded in June and August, respectively: 18.4 and 18.6 °C.

In 2020, the beginning of the growing season had approximate weather conditions as in 2019. April was very dry (Table 5), which negatively affected the tillering of wheat. The rainfall deficiency was compensated for in May and June. These months saw extremely high total waste, respectively: 112.7 and 189.5 mm. Precipitation in July (49.8 mm) was lower compared to the long period average (1951–2021). Precipitation in this period no longer had a major impact on yield. Total monthly precipitation in August (70.1 mm) was similar to the long period average. The highest average daily air temperatures were recorded in August (Figure 6). 

**Figure 7:**
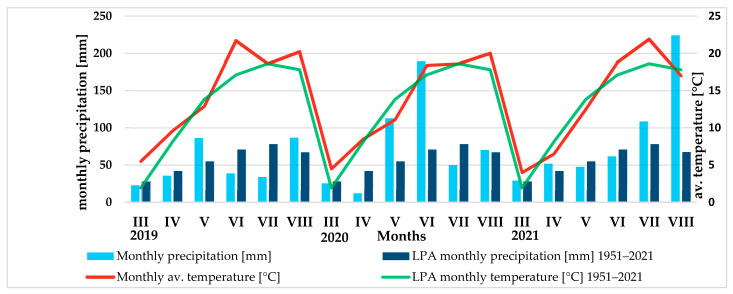
Total precipitation and mean monthly air temperatures for each growing season (2019–2021) compared to the long period average (1951–2021).

In 2021, the most favourable conditions prevailed for the initial growth and development of wheat. In this year, higher rainfall was recorded in March (29 mm) and April (51.7 mm) than in previous years (Figure 6). Based on the Selyaninov index values, weather conditions during these months were defined as being humid and fairly humid, respectively (Table 5). Less favourable meteorological conditions prevailed in May and June. These months were characterised as being fairly dry on the basis of the Selyaninov index values. The total precipitation in May and June was, respectively, 47.4 mm and 61.5 mm. Unfavourable meteorological conditions at this time resulted in poor ear formation, a reduction in the number of grains per ear and, at milk maturity, poor grain formation and a reduction in grain yield. Rainfall in August 2021 was almost three times higher than in the other two years of the study and compared to the long period average. Such conditions caused delayed ripening, grain sprouting, and increased fungal disease infestation. The highest daily temperatures of the year were recorded in July, and in August they were close to the daily means of the long period (Figure 7).

### 3.3. Yield Assessment 

Wheat grain was harvested mechanically with a plot harvester in each year in the first decade of August at full grain maturity (BBCH 85). After harvesting, the grain yield per unit area was determined and given as t ha^−1^.

### 3.4. Grain Physical Properties 

The scope of the tests included the assessment of hectolitre weight (HW) according to EN ISO 7971-3:2019 [69], 1000 grain weight (TGW) [48], grain selectivity (GS), grain uniformity (GU) [48], and grain vitreousness (GV) [48].

### 3.5. Grain Chemical and Rheological Properties 

The scope of the study included the assessment of total ash content (AC) according to AACC 08-01.01 [70], total protein content (PC) according to the Kjeldahl method (N 5.83) on a Kjeltec 8200 (Foss, Hillerød, Sweden) according to the AACC method 46-11.02 [70], wet gluten (WG) and gluten index (GI) via the Glutomatic 2200 (Perten Instruments, Hägersten, Sweden) according to AACC 38-12.02 [70], and falling number (FN) via the Hagberg–Perten method via the Falling Number 1400 (Perten Instruments, Hägersten, Sweden) according to AACC No. 56-81.03 [70].

### 3.6. Statistical Analysis

All measurements were made in a minimum of three replicates. In order to compare the influence of the studied factors, cultivar (n = 4), farming system (n = 3), year (n = 3), and their interaction effects, on the study traits, the analysis of variance ANOVA was applied, and the mean differences were evaluated using Tukey’s test at the significance level of α = 0.05. Additionally, we used principal component analysis (PCA) in order to determine to what extent the studied samples differed from each other and which of the analysed factors had the greatest influence on them.

## 4. Conclusions

The research presented here is in line with current European Union strategies such as the Green Deal and the European Biodiversity Strategy for 2030, which aim to pursue sustainable agricultural production. Our results indicate that higher yields of spring wheat grain can be obtained with less intensive agrotechnology (the INT farming system) than in the high-input system (the CONV farming system), which can be a suitable feedstock for flour production for baking purposes despite its lower protein content (the INT and ORG farming systems). The study also showed that individual wheat cultivars respond differently to the agrotechnology used in cultivation, so it is important to select the production system individually according to the requirements and production capacity of the cultivar. Of the four wheat cultivars tested, Serenada stood out from the others, which, although characterised by a relatively low yield, had high PC and WG content, regardless of the production system under which it was grown. The results of our research are presented to crop farmers, consultants, and food processors. In the future, we plan to continue them by including winter wheat cultivars in the research as well. 

## Figures and Tables

**Table 1:** The effect of year, cultivar, and farming system on the physical properties of grain.

Source of Variation	HW [kg hl^−1^]	TGW [g]	GS [%]	GU [%]	GV [%]
Year	**	n.s.	n.s.	n.s.	**
2019	77.9 ± 2.99 ^c^	33.3 ± 4.32	40.0 ± 15.6	72.3 ± 7.79	54 ± 13.46 ^b^
2020	73.4 ± 2.30 ^b^	33.2 ± 6.33	37.7 ± 16.55	73.3 ± 5.22	63 ± 7.96 ^c^
2021	71.0 ± 4.36 ^a^	33.2 ± 3.29	42.2 ± 13.24	73.9 ± 7.15	41 ± 14.27 ^a^
Cultivar	**	**	**	**	**
Harenda	76.7 ± 3.15 ^c^	34.7 ± 2.08 ^c^	32.7 ± 11.60 ^a^	75.3 ± 5.06 ^c^	56 ± 15.63 ^b^
Kandela	74.0 ± 2.99 ^b^	31.0 ± 2.56 ^b^	36.1 ± 7.99 ^b^	73.1 ± 4.87 ^b^	48 ± 16.68 ^a^
Mandaryna	75.8 ± 3.41 ^bc^	28.4 ± 2.42 ^a^	31.4 ± 1.96 ^a^	79.8 ± 2.06 ^d^	61 ± 12.25 ^b^
Serenada	69.8 ± 4.38 ^a^	39.0 ± 3.20 ^d^	64.4 ± 4.22 ^c^	64.4 ± 2.30 ^a^	47 ± 11.13 ^a^
Farming system	**	n.s.	**	**	**
ORG	73.0 ± 4.99 ^a^	33.8 ± 4.60	41.6 ± 15.05 ^b^	72.2 ± 5.84 ^a^	46 ± 19.91 ^a^
INT	75.6 ± 4.02 ^b^	33.9 ± 4.35	40.2 ± 16.26 ^b^	75.0 ± 6.74 ^b^	57 ± 10.28 ^b^
CONV	73.8 ± 3.67 ^a^	32.1 ± 5.24	38.1± 15.53 ^a^	72.2 ± 7.45 ^a^	55 ± 10.98 ^b^

Data are presented as means ± standard deviations; n.s.—not significant, ** different letters in columns correspond to significant differences (α = 0.05) between means according to Tukey’s test. Abbreviations: HW—hectolitre weight, TGW—1000 grain weight, GS—grain selectivity, GU—grain uniformity, GV—grain vitreousness, ORG—organic, INT—integrated, and CONV—conventional.

**Table 2:** The effect of year, cultivar, and farming system on the chemical and rheological properties of wheat grain.

Source of Variation	AC [% d.m.].	PC [% d.m.].	WG [%]	GI [-]	FN [s]
Year	n.s.	**	n.s.	**	**
2019	1.97 ± 0.12	14.0 ± 1.76 ^a^	28.9 ± 5.23	91 ± 5.43 ^b^	243 ± 80.47 ^a^
2020	2.11 ± 0.07	13.8 ± 1.23 ^a^	30.7 ± 5.40	86 ± 6.49 ^a^	414 ± 40.50 ^b^
2021	2.00 ± 0.11	15.2 ± 1.37 ^b^	30.6 ± 5.06	91 ± 7.18 ^b^	239± 46.44 ^a^
Cultivar	**	**	**	**	**
Harenda	2.03 ± 0.09 ^b^	14.1 ± 3.59 ^a^	28.8± 5.62 ^a^	95 ± 3.36 ^c^	270 ± 102.79 ^a^
Kandela	2.03 ± 0.16 ^b^	14.0 ± 1.49 ^a^	28.2 ± 4.25 ^a^	85 ± 30.16 ^a^	281± 107.50 ^a^
Mandaryna	1.97 ± 0.11 ^a^	14.0 ± 1.91 ^a^	29.3 ± 4.99 ^a^	88 ± 5.87 ^ab^	321± 112.41 ^b^
Serenada	2.08 ± 0.08 ^c^	15.2 ± 1.28 ^b^	34.0 ± 4.14 ^b^	89 ± 6.14 ^b^	323 ± 64.92 ^b^
Farming system	n.s.	**	**	**	**
ORG	2.03 ± 0.14	13.2 ± 1.23 ^a^	27.5 ± 5.90 ^a^	91 ± 5.72 ^b^	265 ± 106.84 ^a^
INT	2.02 ± 0.09	14.3 ± 1.44 ^b^	29.4 ± 4.35 ^b^	91 ± 6.40 ^b^	303 ± 92.55 ^b^
CONV	2.04 ± 6.31	15.5 ± 1.07 ^c^	33.4 ± 3.40 ^c^	87 ± 7.89 ^a^	324 ± 95.16 ^c^

Data are presented as means ± standard deviations; n.s.—not significant, ** different letters in columns correspond to significant differences (α = 0.05) between means according to Tukey’s test. Abbreviations: AC—ash content, PC—protein content, WG—wet gluten, GI—gluten index, FN—falling number, ORG—organic, INT—integrated, and CONV—conventional.

**Table 3:** List of wheat cultivars tested.

Wheat Cultivars	Abbreviation	Country of Origin	Breeding Company
Harenda	H	Poland	MHR Małopolska Hodowla Roślin sp. z o.o.
Kandela	K		DANKO Hodowla Roślin sp. z o.o.
Mandaryna	M		
Serenada	S		Hodowla Roślin Strzelce sp. z o.o. IHAR Group

**Table 4:** Soil chemical properties and agrotechnical treatments used in the different farming systems.

Specification	Farming System		
	ORG	INT	CONV
Soil properties:			
pH _KCl_	5.65	5.90	5.75
C _org_ (g kg^−1^ of soil)	9.9	8.1	8.1
P _Egner_ (mg kg^−1^ of soil)	40.3	84.8	85.4
K _Egner_ (mg kg^−1^ of soil)	64.0	164.0	134.1
Mg (mg kg^−1^ of soil)	69.3	50.1	41.9
Soil tillage	Mouldboard ploughing		
Crop rotation	Potato, spring wheat + grass clover undersown, two * grass clover, winter wheat	Potato, spring wheat + clover, clover, winter wheat	Winter oilseed rape, winter wheat, spring wheat
Organic fertilisation	Compost (30 t ha^−1^) for potato+ catch crop	Compost (30 tha^−1^) for potato + two * catch crop	Rape straw, winter wheat straw
Mineral fertilisation (kg ha^−1^)	According to the crop requirements, natural P + K fertilisers (42 + 60)	N (85) + P (55) + K (75)	N (140) + P (60) + K (80)
Herbicides	0 *	1 *	2 *
Fungicides	0 *	1 *	2 *
Insecticides	0 *	1 *	1 *
The growth regulator	0 *	1 *	1 *
Harrowing	0 *	0 *	1 *

* frequency.

**Table 5:** Values of the Selyaninov hydrothermal index (k) in each growing season (2019–2021).

Month	Year		
	2019	2020	2021
III	optimal	quite wet	wet
IV	quite dry	very dry	very wet
V	wet	extremely wet	quite dry
VI	very dry	extremely wet	quite dry
VII	very dry	dry	optimal
VIII	optimal	quite dry	extremely wet

## Data Availability

The data presented in this study are available upon request from the first author.

## Appendix A

**Table A1:** Plant protection products used in spring wheat cultivated in different farming systems.

Farming System	Plant Protection Products		
	Herbicides	Fungicides	Insecticides
2019			
Organic	–	–	–
Integrated	–	Prosaro 250EC—0.8 l ha^−1^	Fury 100EW—0.1 l·ha^−1^
Conventional	Klinik Duo 360SL—5.0 l ha^−1^	Capallo 337.5SE—1.5 l ha^−1^	Fury 100EW—0.1 l·ha^−1^
	Mustang Forte 195SE—0.8 l ha^−1^	Prosaro 250EC—0.8 l ha^−1^	–
2020			
Organic	–	–	–
Integrated	–	Delaro 325SC—0.8 l ha^−1^	Decis Mega—0.125 l ha^−1^
Conventional	Zevio—4.0 l ha^−1^	Delaro 325SC—0.8 l ha^−1^	Titan 100EW—0.1 l ha^−1^
	Mustang Forte 195SE—0.8 l ha^−1^	Prosaro 250EC—0.8 l ha^−1^	–
2021			
Organic	–	–	–
Integrated	–	Input 460EC—1.0 l ha^−1^	Sherpa 100EC—0.25 l ha^−1^
Conventional	Zevio—4.0 l ha^−1^	Input 460EC—1.0 l ha^−1^	Sherpa 100EC—0.25 l ha^−1^
	Mustang Forte 195SE—0.8 l ha^−1^	Priaxor—1.5 l ha^−1^	–

## References

[1] Shewry P.R. (2009). Wheat. J. Exp. Bot..

[2] FAOSTAT. https://www.fao.org/.

[3] Łaba S., Cacak-Pietrzak G., Łaba R., Sułek A., Szczepański K. (2022). Food Losses in Consumer Cereal Production in Poland in the Context of Food Security and Environmental Impact. Agriculture.

[4] Biel W., Kazimierska K., Bashutska U. (2020). Nutritional value of wheat, triticale, barley and oat grains. Acta Sci. Pol. Zootech..

[5] Cacak-Pietrzak G. (2008). The use of wheat in various branches of the food industry—Technological requirements. Prz. Zboż. Młyn..

[6] Shewry P.R., Hey S.J. (2015). The contribution of wheat to human diet and health. Food Energy Secur..

[7] FAO (2018). Plant Nutrition for Food Security: A Guide for Integrated Nutrient Management.

[8] Váňová M., Klem K., Míša P., Matušinsky P., Hajšlová J., Lancová K. (2008). The content of Fusarium mycotoxins, grain yield and quality of winter wheat cultivars under organic and conventional cropping systems. Plant Soil Environ..

[9] Dziki D., Cacak-Pietrzak G., Gawlik-Dziki U., Świeca M., Miś A., Różyło R., Jończyk K. (2017). Physicochemical properties and milling characteristics of spring wheat from different farming systems. J. Agric. Sci. Technol..

[10] Marzec A., Cacak-Pietrzak G., Gondek E. (2011). Mechanical and acoustic properties of spring wheat versus its technological quality factors. J. Texture Stud..

[11] Rozbicki J., Ceglińska A., Gozdowski D., Jakubczyk M., Cacak-Pietrzak G., Mądry W., Golba J., Piechociński M., Sobczyński G., Studnicki M. (2015). Influence of the cultivar, environment and managment on the grain yield and bread-making quality in winter wheat. J. Cer. Sci..

[12] Knapowski T., Kozera W., Chmielewski J., Gorczyca D., Wszelaczyńska E., Pobereżny J. (2016). Mineral fertilization as a factor determining technological value of grain of *Triticum aestivum* ssp. *spelta* L.. J. Exp. Agric. Int..

[13] Mäder P., Hahn D., Dubois D., Gunst L., Alföldi T., Bergmann H., Oehme M., Amadò R., Schneider H., Graf U. (2007). Wheat quality in organic and conventional farming: Results of 21-year field experiment. J. Sci. Food Agric..

[14] Zargar M., Polityko P., Pakina E., Bayat M., Vandyshev V., Kavhiza N., Kiselev E. (2018). Productivity, quality and economics of four spring wheat (*Triticum aestivum* L.) cultivars as affected by three cultivation technologies. Agron. Res..

[15] Sułek A., Cacak-Pietrzak G. (2018). The influence of production technology on yield selected quality parameters of spring wheat cultivars. Agric. Sci. Crop Sci. Animal Sci. Res. Rural Develop..

[16] Sułek A., Cacak-Pietrzak G., Wyzinska M., Nieróbca A. (2019). Influence of nitrogen fertilization on the yields and grain quality of winter wheat under different environmental conditions. Int. J. Agric. Biol. Eng..

[17] Minhas W.A., Mehboob N., Yahya M., Rehman H.U., Farooq S., Hussain M. (2023). The Influence of Different Crop Mulches on Weed Infestation, Soil Properties and Productivity of Wheat under Conventional and Conservation Production Systems. Plants.

[18] Gawęda D., Haliniarz M. (2021). Grain Yield and Quality of Winter Wheat Depending on Previous Crop and Tillage System. Agriculture.

[19] Yousefian M., Shahbazi F., Hamidian K. (2021). Crop Yield and Physicochemical Properties of Wheat Grains as Affected by Tillage Systems. Sustainability.

[20] Aula L., Easterly A.C., Creech C.F. (2022). Winter Wheat Seeding Decisions for Improved Grain Yield and Yield Components. Agronomy.

[21] Cristache S.-E., Vuță M., Marin E., Cioacă S.-I., Vuţă M. (2018). Organic versus Conventional Farming—A Paradigm for the Sustainable Development of the European Countries. Sustainability.

[22] Buráňová S., Černý J., Mitura K., Lipińska K.K., Kovářík J., Balík J. (2016). Effect of organic and mineral fertilizers on yield parameters and quality of wheat grain. Sci. Agric. Bohem..

[23] Krejčířová L., Capouchová I., Petr J., Bicanová E., Faměra O. (2007). The effect of organic and conventional growing systems on quality and storage protein composition of winter wheat. Plant Soil Environ..

[24] Durham T.C., Mizik T. (2021). Comparative Economics of Conventional, Organic, and Alternative Agricultural Production Systems. Economies.

[25] McArthur J.W., McCord G.C. (2017). Fertilizing growth: Agricultural inputs and their effects in economic development. J. Dev. Econ..

[26] Michalczyk J. (2012). Food security in the face of globalization. Ekonomia.

[27] Fischer R.A., Byerlee D., Edmeades G.O. (2014). Crop Yields and Global Food Security: Will Yield Increase Continue to Feed the World? ACIAR Monograph No. 158.

[28] UN, Summary of Results World Population Prospects 2022. https://www.un.org/development/desa/pd/sites/www.un.org.development.desa.pd/files/wpp2022_summary_of_results.pdf.

[29] Ponti T.D., Rijk B., Ittersum M.K. (2012). The crop yield gap between organic and conventional agriculture. Agric. Syst..

[30] Billsborrow B., Tétard-Jones C., Średnicka-Tober D., Cooper J. (2013). The effect of organic and conventional management on the yield and quality of wheat grown in a long-term field trial. Eur. J. Agron..

[31] Mazzoncini M., Antichi D., Silvestri N., Ciantelli G., Sgherri C. (2015). Organically vs conventionally grown winter wheat: Effects on grain yield, technological quality, and on phenolic composition and antioxidant properties of bran and refined flour. Food Chem..

[32] Głodowska M., Gałązka A. (2018). Unsustainable agriculture and its environmental consequences. Zesz. Probl. Post. Nauk Roln..

[33] Wrzaszcz W., Prandecki K. (2020). Agriculture and the European Green Deal. Probl. Agric. Econ..

[34] Ben Hassen T., El Bilali H. (2022). Impacts of the Russia-Ukraine War on Global Food Security: Towards More Sustainable and Resilient Food Systems?. Foods.

[35] Linina A., Ruža A. (2008). The influence of cultivar, weather conditions and nitrogen fertilizer on winter wheat grain yield. Agron. Res..

[36] Holík L., Hlisnikovský L., Kunzová E. (2018). The effect of mineral fertilizers and farmyard manure on winter wheat grain yield and grain quality. Plant Soil Environ..

[37] Cesevičienė J., Leistrumaitė A., Paplauskienė V. (2009). Grain yield and quality of winter wheat varieties in organic agriculture. Agron. Res..

[38] Polityko P., Rebouh N.Y., Kucher D., Vvedenskiy V., Kapranov V., Atmachian G., Behzad A., Urazova E., Khomenets N., Pakina E. (2020). Productivity and grain quality of three spring wheat (*Triticum aestivum* L.) cultivars under three cultivation technologies. EurAsian J. BioSciences.

[39] Kuś J., Jończyk K., Stalenga J., Feledyn-Szewczyk B., Mróz A. (2011). Yields of the selected spring wheat varieties cultivated in organic and conventional crop production systems. J. Agric. Eng..

[40] Feledyn-Szewczyk B., Cacak-Pietrzak G., Lenc L., Stalenga J. (2020). Rating of Spring Wheat Varieties (*Triticum aestivum* L.) According to Their Suitability for Organic Agriculture. Agronomy.

[41] Chmura K., Chylinska E., Dmowski Z., Nowak L. (2009). Role of the water factor in yield formation of chosen field crops. Infrastruct. Ecol. Rural. Areas.

[42] Fowler D.B. (2003). Crop Nitrogen Demand and Grain Protein Concentration of Spring and Winter Wheat. Agron. J..

[43] Koppel R., Ingver A. (2008). A comparison of the yield and quality traits of winter and spring wheat. Latv. J. Agron..

[44] Studnicki M., Wijata M., Sobczyński G., Samborski S., Rozbicki J. (2018). Assessing grain yield and quality traits stability of spring wheat cultivars at different crop management levels. Cereal Res. Commun..

[45] Kołodziejczyk M., Szmigiel A., Oleksy A. (2007). Influence of cultivation intensity on yielding of selected varieties of spring wheat. Acta Sci. Pol. Agric..

[46] Szymona J. (2011). Evaluation of spring wheat varieties in the aspect of organic production’s usefulness. J. Agric. Eng. Res..

[47] Švančárková M., Žák S. (2015). The grain quality of winter wheat in organic and conventional farming. Acta Fytotech. Zootech..

[48] Cacak-Pietrzak G. (2011). Studies on the Effect of Ecological and Conventional System of Plant Production on the Technological Value of Selected Varieties of Winter Wheat, Treatises and Monographs.

[49] Sobolewska M., Stankowski S. (2017). The influence of farming systems on the technological quality of grain and flour cultivars of winter wheat. Folia Pomer. Univ. Technol. Stetin. Agric. Aliment. Pisc. Zootech..

[50] (1997). Cereal Grain—Common Wheat.

[51] Dziki D., Cacak-Pietrzak G., Miś A., Jończyk K., Gawlik-Dziki U. (2014). Influence of wheat kernel physical properties on the pulverizing process. J. Food Sci. Technol..

[52] Feledyn-Szewczyk B., Cacak-Pietrzak G., Lenc L., Gromadzka K., Dziki D. (2021). Milling and Baking Quality of Spring Wheat (*Triticum aestivum* L.) from Organic Farming. Agriculture.

[53] Nazarenko M., Mykolenko S. (2020). Variation in grain productivity and quality of modern winter wheat varieties in northern Ukrainian Steppe. Ukr. J. Ecol..

[54] Mazurkiewicz J. (2005). Comparison of the technological quality of wheat and rye grown in conventional and organic farm conditions. Acta Agrophysica.

[55] Kwiatkowski C., Wesołowski M., Harasim E., Kubecki J. (2006). Yield and grain quality of winter wheat cultivars depending on the level of agricultural technology. Pam Puławski.

[56] Brankovic G., Dodig D., Zorić M., Surlan-Momirovic G., Dragicevic V., Djuric N. (2014). Effects of climatic factors on grain vitreousness stability and heritability in durum wheat. Turk. J. Agric. For..

[57] Mazzoncini M., Belloni P., Risaliti R., Antichi D. Organic Vs Conventional Winter Wheat Quality and Organoleptic Bread Test. Proceedings of the 3rd QLIF Congress.

[58] Kihlberg I., Öström Å., Johannson L., Risvik E. (2006). Sensory qualities of plain white pan bread: Influence of farming system, year of harvest and baking technique. J. Cer. Sci..

[59] COBORU. https://coboru.gov.pl/PlikiWynikow/48_2021_WPDO_7_PSZJ.pdf.

[60] Toader M., Georgescu E., Nastase P.I., Ionescu A.M. (2019). Some aspects of bakery industry quality for organic and conventional wheat. Sci. Pap. Ser. A. Agron..

[61] Woźniak A., Rachoń L., Stępniewska A. (2017). Spring wheat grain quality in relations to a cropping system. J. Elem..

[62] Łysoń E., Biel W., Sobolewska M. (2015). Estimation of the selected winter wheat (*Triticum aestivum* L.) varieties cultivated in organic and conventional crop production systems. Folia Pomer. Univ. Technol. Stetin. Agric. Aliment. Pisc. Zootech..

[63] Oleksy A., Szmigiel A., Kołodziejczyk M. (2008). Influence of cultivation intensity on protein content and yield of winter wheat cultivars. Acta Sci. Pol. Agric..

[64] Keres I., Alaru M., Koppel R., Altosaar I., Tosens T., Loit E. (2021). The Combined Effect of Nitrogen Treatment and Weather Conditions on Wheat Protein-Starch Interaction and Dough Quality. Agriculture.

[65] Ingver A., Tamm I., Tamm Ü. (2008). Effect of Organic and Conventional Production on Yield and the Quality of Spring Cereals. Latv. J. Agron..

[66] Biel W., Maciorowski R. (2012). Evaluation of the nutritional value of selected wheat varieties. Food Sci. Technol. Qual..

[67] Linina A., Ruža A. Impact of agroecological conditions on the Hagberg falling number of winter wheat grain. Proceedings of the Annual 21st International Scientific conference: Research for Rural Development.

[68] Skowera B. (2014). Changes in hydrothermal conditions in Poland (1971−2010). Fragm. Agron..

[69] (2019). Cereals: Determination of Bulk Density, Called Mass Per Hectoliter—Part 3: Routine Method.

[70] AACC (2010). Method 08-01.01: Total ash., Method 46-11.02: Crude Protein—Improved Kjeldahl Method, Method 38-12.02: Wet gluten and Gluten Index, Method 56-81.03: Determination of Falling Number. Official Methods of the American Association of Cereal Chemists.

